# Optimising sleep and managing stress by integrating a behavioural intervention during cardiac rehabilitation: rationale and design of the OPTICARE-RESST multicentre randomised controlled trial

**DOI:** 10.1136/bmjopen-2026-117963

**Published:** 2026-04-16

**Authors:** Inge van Loon, Esmée Ooms, Annemarie Luik, Madoka Sunamura, Tamara Wally, Eric Boersma, Tom Vromen, Nicole Tenbült-van Limpt, Hans Bussmann, Mariette Boon, Maartje Louter, Ken Redekop, Erwin Ista, Rita van den Berg-Emons, Nienke ter Hoeve, on behalf of the other OPTICARE-RESST Consortium members, Arnoud van 't Hof

**Affiliations:** 1 Department of Rehabilitation Medicine, Erasmus MC University Medical Center Rotterdam, Rotterdam, The Netherlands; 2 Capri Cardiac Rehabilitation, Rotterdam, The Netherlands; 3 The Netherlands Institute of Mental Health and Addiction, Trimbos Institute, Utrecht, The Netherlands; 4 Cardiovascular Institute, Department of Cardiology, Erasmus MC University Medical Center Rotterdam, Rotterdam, The Netherlands; 5 Department of Cardiology, Máxima Medical Centre, Eindhoven/Veldhoven, The Netherlands; 6 Department of Internal Medicine, Division of Endocrinology, Erasmus MC University Medical Center Rotterdam, Rotterdam, The Netherlands; 7 Obesity Center CGG, Department of Internal Medicine, Division of Endocrinology, Erasmus MC University Medical Center Rotterdam, Rotterdam, The Netherlands; 8 Department of Neurology, Erasmus MC University Medical Center Rotterdam, Rotterdam, The Netherlands; 9 Erasmus School of Health Policy and Management, Erasmus University Rotterdam, Rotterdam, The Netherlands; 10 Department of Internal Medicine, Section Nursing Science, Erasmus MC University Medical Center Rotterdam, Rotterdam, The Netherlands; 11 Department of Neonatal and Pediatric Intensive Care, Division of Pediatric Intensive Care, Erasmus MC Sophia Children’s Hospital, University Medical Centre Rotterdam, Rotterdam, The Netherlands

**Keywords:** Stress, Psychological, Randomized Controlled Trial, Cardiovascular Disease, REHABILITATION MEDICINE, SLEEP MEDICINE

## Abstract

**Introduction:**

Over 50% of patients participating in cardiac rehabilitation (CR) experience poor sleep and/or, closely related, psychological stress. Although stress management interventions are generally available, they are typically underutilised in CR, and sleep remains an underaddressed component within CR. This is concerning, as poor sleep and stress not only reinforce each other but are also associated with poorer cardiovascular health and lower quality of life. Therefore, the primary aim of the OPtimising CArdiac REhabilitation by REfining Sleep and STress (RESST) study is to investigate the (cost-)effectiveness of adding a behavioural intervention targeted at improving sleep and managing stress during CR (RESST intervention) on sleep and psychological stress. Furthermore, the study aims to explore the (bidirectional) associations between sleep, stress and lifestyle behaviours.

**Methods and analysis:**

This parallel-arm multicentre randomised controlled trial will include 200 CR patients across 3 major CR centres in the Netherlands who experience poor sleep and/or stress. Patients will be randomised in a 1:1 ratio to standard CR or standard CR with the RESST intervention. Standard CR is a structured programme combining exercise, lifestyle guidance and risk management. On top of standard CR, the RESST intervention consists of 5 in-person group sessions targeting sleep and stress and is based on Acceptance and Commitment Therapy and Cognitive Behavioural Therapy. Primary outcomes are accelerometer-assessed and self-reported sleep and perceived stress. Secondary outcomes include quality of life, psychosocial well-being, chronic stress biomarkers (hair cortisol and cortisone), momentary fatigue, momentary stress and physical activity. Linear mixed models will be used to assess changes in outcomes at 3-month (after intervention and/or CR completed) and 6-month follow-up. The momentary data collected with ecological momentary assessment and accelerometry will be analysed using multilevel linear mixed models to explore the (bidirectional) relationship between sleep, stress and other lifestyle components such as physical activity.

**Ethics and dissemination:**

This study was approved by the ethics committee of Erasmus MC, Erasmus University Medical Centre, Rotterdam, the Netherlands (MEC-2024-0238). The findings will be disseminated through publications in peer-reviewed journals, presentations at academic conferences and professional and patient publications.

**Trial registration number:**

NCT06505109.

**Strengths and limitations of this study**

- Combination of objective and self-reported assessment of the primary outcomes, sleep and stress, to minimise bias and provide multifaceted insight into these factors.
- Use of ecological momentary assessment to determine associations in a daily life context between sleep, stress and lifestyle behaviours such as physical activity and diet.
- The REfining Sleep and STress intervention is aligned with existing cardiac rehabilitation practices, enhancing the potential for future implementation in standard care.
- Study participants cannot be blinded due to the behavioural nature of the intervention.
- Follow-up time is 6 months, limiting the possibility to assess long-term cardiovascular health outcomes.

## Introduction

 Cardiac rehabilitation (CR) focuses on the secondary prevention of cardiovascular disease through a multidisciplinary approach that includes exercise training, psychosocial counselling and lifestyle education and modification.^1^ CR was shown to reduce mortality and hospital (re)admissions, and to improve quality of life (QoL) in a cost-effective manner for cardiac patients.^1^,^5^ However, despite these benefits, 10%–30% of patients experience recurrent cardiovascular events or require cardiac surgery after completing CR, indicating the need for further optimisation.^5 6^

One area with clear potential for further improvement is sleep, as this is generally unaddressed in current CR programmes. Yet, more than half of cardiac patients experience poor sleep,^7 8^ with one study reporting numbers as high as 87%.^9^ In comparison, it is estimated that around one-third of the general population experiences poor sleep.^10^,^12^ Poor sleep has been associated with lower cardiovascular health and a 50% increased risk of a recurrent cardiac event,^13 14^ as well as lower QoL, lower treatment adherence and lower self-efficacy, which may impede the effectiveness of CR.^7 15^

Similar to sleep, and highly interconnected,^16^ psychological stress is also widely present in patients with cardiovascular disease. A large meta-analysis estimated that psychological stress affects 58% of all cardiac patients.^17^ Moreover, long-term stress has been shown to predict future cardiovascular disease.^18^ Stress management programmes are often available during CR^1^ and have been shown to improve perceived stress, anxiety and depression and reduce cardiovascular event rates by 50%.^19 20^ However, stress management training during CR is often underused,^21 22^ leaving patients without the support they may need.

Despite the strong interconnection between sleep and stress, interventions that target only sleep or only stress are often insufficient to optimally improve either. A study has shown that 85% of CR participants following stress management training experienced poor sleep, and that the training was less effective in improving stress in those patients with insomnia.^23^ Similarly, long-term improvements in insomnia severity after cognitive behavioural therapy for insomnia (CBT-i) were less likely to be sustained among those who also experienced stress.^24^ These findings suggest that combined targeting of sleep and stress may be required to effectively address these often co-occurring complaints.

Beyond assessing the effectiveness of interventions addressing both sleep and stress, it is important to explore the underlying mechanisms by examining how these factors interact. Accumulating evidence indicates a bidirectional relationship between sleep and stress.^25^ Day-to-day studies in healthy adults, for example, showed that higher evening stress predicts shorter subsequent sleep, while poor sleep increases next-day stress.^26^ Sleep and stress may also interact with other lifestyle behaviours central to CR. For example, it has been suggested that daytime physical activity can influence sleep quality, potentially moderated by perceived stress,^27 28^ whereas sleep during the night can influence physical activity levels the following day.^29^ Last, parameters of diversity, such as sex, age, ethnicity and socioeconomic status, may also influence the effectiveness of interventions.^16 30^ A better understanding of how lifestyle factors and diversity parameters are intertwined with sleep and stress can be used to optimise and tailor behavioural interventions for sleep and stress, as well as CR programmes.

The primary aim of the OPtimising CArdiac REhabilitation by REfining Sleep and STress (OPTICARE-RESST) trial is to determine the effectiveness of integrating a behavioural programme targeting sleep and stress simultaneously (RESST-intervention) into CR. We hypothesise that the intervention will not only improve sleep and stress, but also improve cardiometabolic health, lifestyle behaviours and psychosocial well-being. Moreover, we expect this intervention to be cost-effective as it may reduce (mental) healthcare uptake, hospital admissions and mortality, while improving QoL. Secondary aims are to examine the bidirectional relationships between sleep, stress and lifestyle factors to get more insight into the working mechanisms; determine whether aspects of diversity are associated with intervention effectiveness; and identify barriers and enablers of the intervention relevant to implementation in practice.

## Methods and analysis

### Design

**Figure 1:**
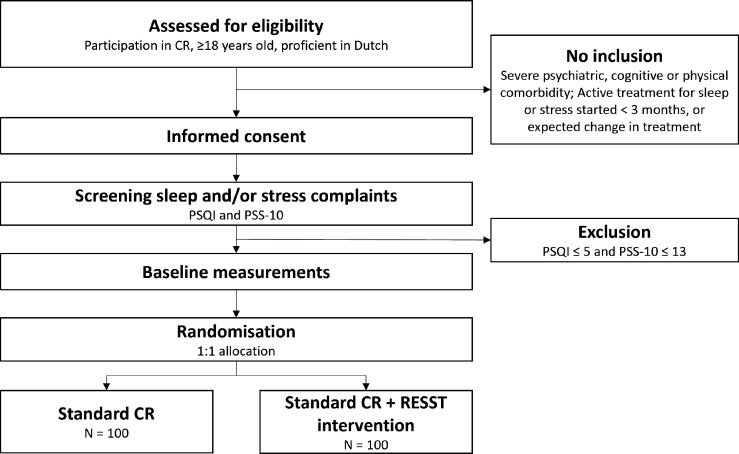
Flow chart of the study inclusion process. CR, cardiac rehabilitation; PSQI, Pittsburgh Sleep Quality Index; PSS-10, 10 item Perceived Stress Scale; RESST, REfining Sleep and STress.

OPTICARE-RESST is a multicentre randomised controlled trial (RCT). Patients will be recruited from three large CR centres across the Netherlands: Capri Cardiac Rehabilitation in Rotterdam and in The Hague, and Máxima Medical Centre in Veldhoven/Eindhoven. Participants are randomly allocated in a 1:1 ratio to a 6–12 week standard CR programme (control group), or a standard CR programme with an additional behavioural intervention to improve sleep and manage stress (intervention group). A total of 200 participants will be included (figure 1). All participants need to provide signed informed consent before study entry (online supplemental material). Data are collected at the start of CR (baseline), at 3 months (post-CR) and at 6 months.

The study was registered at ClinicalTrials.gov (NCT06505109) prior to trial initiation. The protocol was written using Standard Protocol Items: Recommendations for Interventional Trials reporting guidelines (online supplemental material).^31^ Ethical approval was obtained from the ethics committee of Erasmus MC, Erasmus University Medical Centre, Rotterdam, the Netherlands (MEC-2024-0238).

### Patient population and randomisation

To be eligible for inclusion, participants need to (1) participate in CR at one of the study centres for any cardiac diagnosis or reason as stated in the Dutch guidelines,^1^ (2) be at least 18 years of age, (3) be proficient in the Dutch language, (4) experience poor sleep (Pittsburgh Sleep Quality Index (PSQI) >5^32^) or psychological stress (Perceived Stress Scale-10 (PSS-10) >13^33 34^) and (5) be willing to provide signed informed consent before study entry. Exclusion criteria are (1) having a severe psychiatric, cognitive or physical comorbidity that would impede CR participation and (2) active treatment for sleep disorders, stress or other forms of (behavioural) therapy at the start of the study or expected to start within 6 months that could interfere with the RESST intervention. Participants who have received prior treatment targeting sleep, stress or a related complaint (eg, continuous positive airway pressure users or sleep medication), that is still ongoing and has resulted in symptom stability in the past 3 months, are eligible.

Participants who fulfil inclusion and exclusion criteria are randomised (1:1) to the control or intervention group after baseline measurements are completed. Randomisation is performed using Castor software, which generates a random allocation sequence in variable blocks of 2, 4 and 6, stratified by participating centres. First patient inclusion took place on 28 August 2024 and is expected to last for a period of 2 years.

### Control group

The control group will receive standard CR. Participating centres adhere to the Dutch CR guidelines,^1^ which are largely in line with the guidelines of the European Society of Cardiology,^35^ as well as the guidelines of the American Heart Association.^36^ Standard CR programmes consist of 6–12 weeks of twice-weekly supervised, group-based exercise sessions and educational sessions on cardiovascular risk factors, living with cardiovascular disease and medication use, healthy lifestyle and coping. The duration of the CR programme is based on the needs and complexity of the patient. Group counselling is available when indicated e.g., on stress management or relaxation, and a healthy diet, as well as individual guidance from a social worker or occupational therapist, psychologist or dietitian. Referral for smoking cessation is possible if needed.

### Intervention group

**Figure 2:**
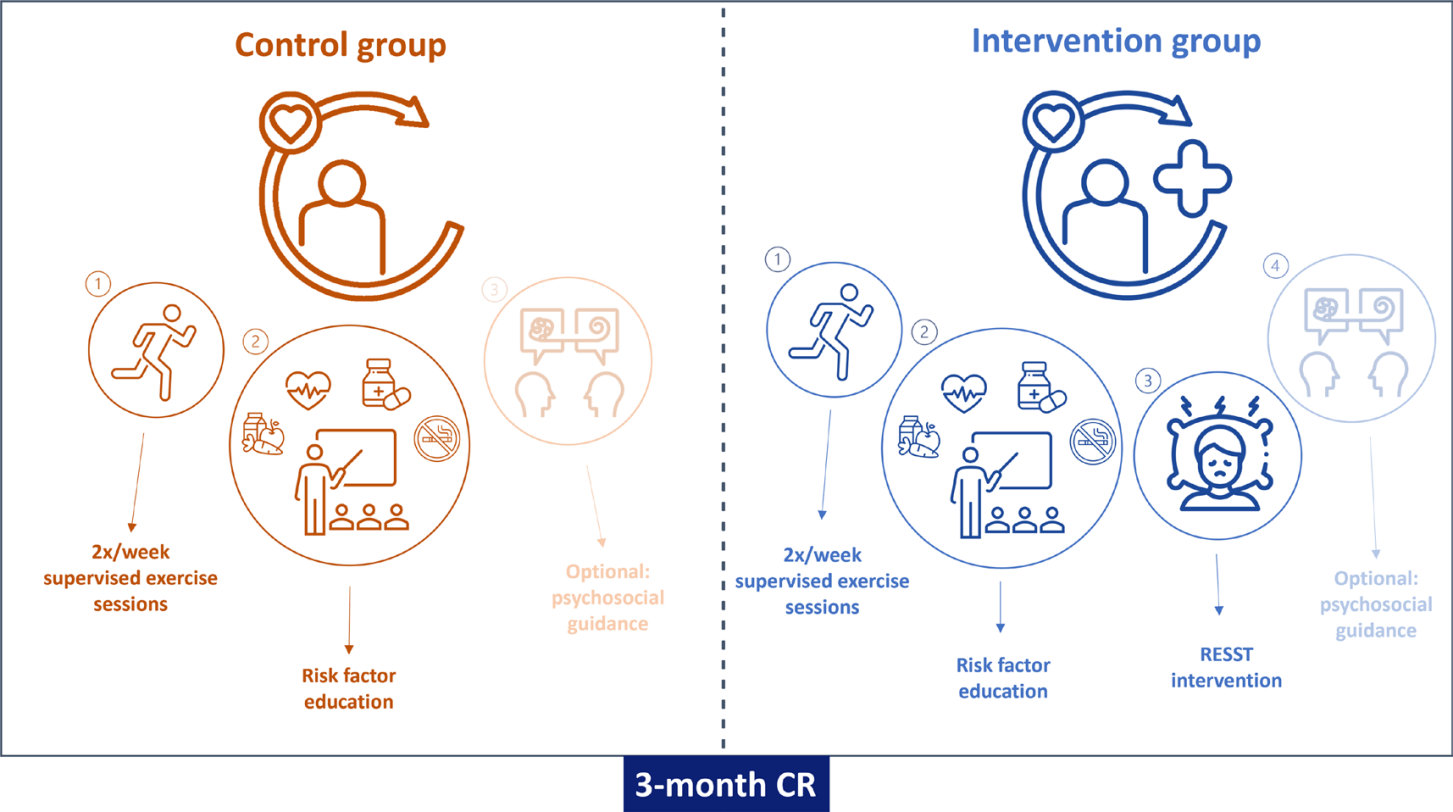
Study components of the control and the intervention (RESST) group. CR, cardiac rehabilitation; RESST, REfining Sleep and STress.

The intervention group receives the RESST intervention in addition to standard CR (figure 2). The RESST intervention consists of five group sessions lasting 120 min each, under the guidance of a trained psychologist or social worker.

The intervention was developed at Capri Cardiac Rehabilitation and is based on principles from Acceptance and Commitment Therapy (ACT) and ACT for insomnia (ACT-i), where the observation and acceptance of thoughts, emotions and physical experiences is a central concept, while committing to value-based actions.^37^ Additionally, elements of CBT-i are incorporated, including psychoeducation on sleep and its underlying physical processes, sleeping conditions and stress reduction.

The RESST intervention takes a comprehensive approach (table 1) to address sleep-related difficulties and stress management, incorporating the following elements:

- Psychoeducation: Participants receive education about the physiological aspects of sleep and the interplay between sleep, stress management and overall well-being and health. They learn about the importance of sleep hygiene (including stimulus control, and if necessary: sleep restriction) and stress reduction in promoting better sleep and (mental) health.
- Mindfulness and cognitive defusion: Mindfulness exercises are practised, cultivating awareness of present-moment experiences. Participants learn to observe and accept thoughts, emotions and physical sensations related to sleep and stress without becoming entangled in them. They are guided to observe their thoughts without automatically reacting to them. By developing awareness of unhelpful thoughts and deeply ingrained beliefs and creating distance from them, individuals can reduce their impact on emotions and behaviours.
- Acceptance and self-compassion: Participants learn to make space for inner discomfort (difficult thoughts, negative emotions and unpleasant physical sensations), without having to change them or be limited by them. They gain insight into self-imposed pressure and develop a kind, supportive attitude towards themselves.
- Values clarification and committed action: Through guided exercises within and between sessions, participants identify their personal values and goals related to sleep, stress management and overall health. This process aids them in aligning their behaviours with their values and establishing a sense of purpose in making positive changes.

**Table 1:** Components of the RESST intervention

Session	Topics discussed
Week 1	Biological mechanisms and functions of stress; Effects of long-term stress; Interaction between sleep and stress; Recognising stress signals and behaviours;
Week 2	Biological mechanisms and functions of sleep; Sleep hygiene and lifestyle factors; Insight into own sleeping habits; Insights in thoughts in daily situations;
Week 3	Recognising own patterns around sleep; Role of thoughts when tense; Observing thoughts; Behaviour when lying awake;
Week 4	Recognising own thought patterns; Time-outs for recognising stress signals; Mindfulness; Role of thoughts in coping with stress; Making healthier decisions in daily life regarding stress and relaxation; Acceptance; Letting go of built-up tension; Cognitive defusion;
Week 5	Value clarification and committed action; Making healthier decisions in daily life regarding stress and relaxation; Mindfulness; Recognising physical tension and letting go; Acceptance; Self-compassion.

RESST, REfining Sleep and STress.

Between each session, participants receive handouts with information and exercises to reflect on their own behaviour patterns and practice their skills between sessions.

### Outcome assessment

**Figure 3:**
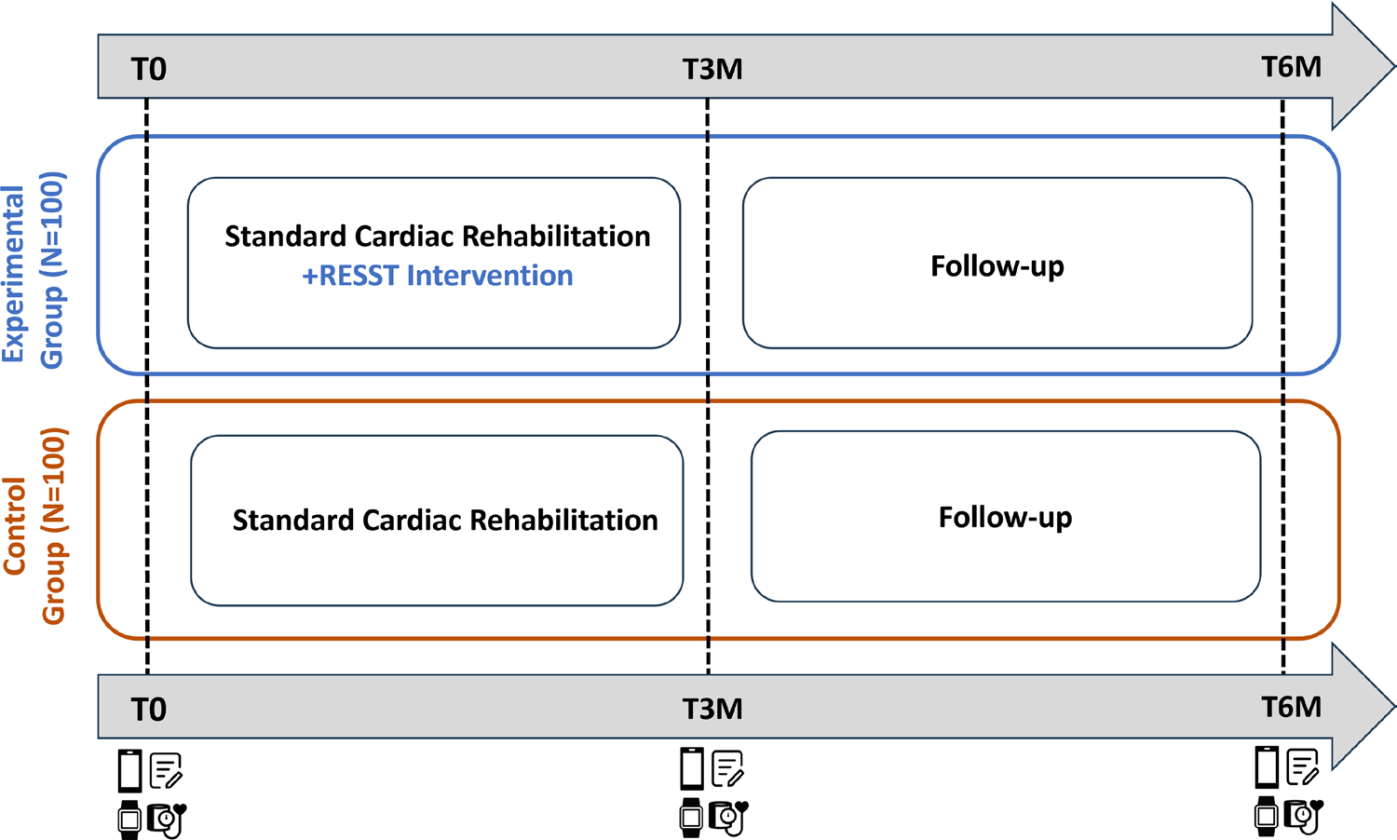
Timeline of the study showing follow-up duration and timing of the measurements (physical examination, questionnaires, accelerometer, EMA). EMA, ecological momentary assessment; RESST, REfining Sleep and STress.

Baseline characteristics and measurements are collected at the start of the CR programme, 3 months later (post-CR) and a final assessment is performed 6 months after the start of CR (figure 3). An overview of all outcomes, manner and timing of assessment is shown in table 2. Below, we elaborate on the primary and secondary outcomes and their manner of assessment.

**Table 2:** Outcomes, manner of assessment and timing of assessment

Outcome	Measurement tool	Assessment
Primary outcomes		
Accelerometry-assessed sleep (sleep duration, sleep latency, wake-after-sleep-onset and sleep efficiency)	GENEActiv accelerometer (Activinsights, Kimbolton, UK)	T0, T3m, T6m
Self-reported sleep quality	Pittsburgh Sleep Quality Index^32^	T0, T3m, T6m
Perceived stress level	Perceived Stress Scale-10^41^	T0, T3m, T6m
Secondary outcomes		
Cardiometabolic risk factors		
Biomarkers of chronic stress: hair cortisol and cortisone	Liquid chromatography-tandem mass spectrometer (Waters, Manchester, UK)	T0, T3m, T6m
Body mass index	Stadiometer, weighing scale	T0, T3m, T6m
Blood pressure	Automatic sphygmomanometer (Omron X2 Basic)	T0, T3m, T6m
Smoking behaviour	Carbon monoxide breath tester (piCO Smokerlyzer, Bedfont Scientific, Maidstone, UK) and self-designed questionnaire	T0, T3m, T6m
Physical fitness (hand-grip strength)	Hand-held dynamometer (Jamar)	T0, T3m, T6m
Physical activity and sedentary behaviour	GENEActiv accelerometer	T0, T3m, T6m
Estimated cardiorespiratory fitness	FitMáx-Questionnaire^63^	T0, T3m, T6m
Alcohol use	Five Shot questionnaire^64^	T0, T3m, T6m
Psychosocial measures		
Health-related quality of life	MacNew^65^	T0, T3m, T6m
Fatigue	Fatigue Severity Scale^66^	T0, T3m, T6m
Anxiety and depression	Hospital Anxiety and Depression Scale^67^	T0, T3m, T6m
Participation in society	Utrechtse Schaal voor Evaluatie van Revalidatie-Participatie^68^	T0, T3m, T6m
Sleep and stress disorders		
Presence of restless legs syndrome	Restless Leg Syndrome Rating Scale^69^	T0
Presence of insomnia	Insomnia Severity Index^70^	T0
Risk of presence of obstructive sleep apnoea	STOP-Bang questionnaire^71^	T0
Diagnosis of sleep disorders	EHR and anamneses	T0
Prior treatment for sleep disorders	EHR and anamneses	T0
Prior treatment for stress management	EHR and anamneses	T0
Cost-effectiveness and implementation parameters		
Costs incurred by patients	iMTA Medical Consumption Questionnaire,^72^ iMTA Productivity Costs Questionnaire,^73^ EHR, costs from the intervention	T3m, T6m
Health utility score	EuroQol 5D-5L (EQ-5D-5L)^74^	T0, T3m, T6m
Satisfaction RESST intervention	Self-designed questionnaire	T3m
Facilitators and enablers of the intervention	Semi-structured interviews with patients and healthcare professionals	Post-intervention
Momentary measures		
Perceived sleep	Electronic diary: Consensus Sleep Diary (CSD)^46^	T0, T3m, T6m
Diet	Electronic diary: Intake of caffeine and alcohol, timing of the main meal (self-designed)	T0, T3m, T6m
Momentary fatigue	Electronic diary: self-designed questions	T0, T3m, T6m
Momentary stress	Electronic diary: self-designed questions	T0, T3m, T6m
Adherence		
Adherence to CR treatment	EHR	T3m
Adherence to the RESST intervention	EHR	T3m
Baseline characteristics		
Sex	Single-item question	T0
Age	EHR	T0
Cardiac diagnosis	EHR	T0
(Changes in) Medication use	EHR	T0, T3m, T6m
Drug use	Self-designed questionnaire	T0
Socioeconomic status	Neighbourhood socioeconomic status determined via postal code (EHR)	T0
Country of origin	Single item question	T0
Country of origin of parents	2-item question	T0
Employment status and presence of irregular or night shifts	2-item question	T0
Marital status	Single-item question	T0
Educational level	Single-item question	T0

CR, Cardiac rehabilitation; EHR, electronic health record; RESST, REfining Sleep and STress; T0, baseline assessment; T3m, 3-month assessment; T6m, 6-month assessment.

#### Primary outcomes

The primary outcomes are both accelerometer-assessed and self-reported sleep, and perceived psychological stress. Sleep is objectively assessed using the GENEActiv accelerometer, which is worn on the non-dominant wrist for 7 consecutive days and nights. The GENEActiv is a tri-axial, wrist-worn accelerometer, which has shown good agreement for sleep-related parameters with gold-standard polysomnography.^38^ With validated algorithms (GGIR package in R), sleep duration, sleep latency, wake-after-sleep-onset and sleep efficiency are estimated. Sleep latency is the time it takes to fall asleep, wake-after-sleep-onset is the time spent awake between the first time falling asleep and the last time waking up, and sleep efficiency is the percentage of time spent asleep between the first time falling asleep and last awakening. Additionally, sleep quality is self-reported with the PSQI.^32^ The PSQI is a widely used tool to measure sleep quality, also in cardiac populations, and was shown to be valid in both clinical and non-clinical samples.^39 40^ Psychological stress is measured with the PSS-10, which was shown to be valid for assessing perceived stress.^41 42^

#### Secondary outcomes

Secondary outcomes include cardiometabolic risk factors, including physical behaviour, biomarkers of chronic stress, blood pressure, body mass index, physical fitness and smoking behaviour. Physical behaviour, comprising physical activity and sedentary behaviour, is assessed using the GENEActiv accelerometer. Using a liquid chromatography-tandem mass spectrometer, biomarkers for chronic stress (cortisol and cortisone) are determined from the most proximal centimetre of a scalp hair sample. During each study visit, blood pressure is measured twice in a sitting position using an automatic sphygmomanometer and averaged. Height and weight are measured without shoes and with heavy garments or items removed. Hand-grip strength, a measure of physical fitness, is assessed using a hand-held dynamometer. Smoking behaviour is tested with a carbon monoxide breathalyser.

Questionnaires are used to assess psychosocial outcomes, the possible presence of sleep disorders, including the risk of having obstructive sleep apnoea, alcohol and smoking behaviour, treatment satisfaction in the intervention group, QoL, and intervention, medical and productivity costs incurred for the cost-effectiveness analysis (table 2). Semistructured interviews with patients and their relatives, as well as healthcare providers, will be conducted to gather information on enablers and barriers of the RESST intervention.

Momentary outcomes are assessed using smartphone-based ecological momentary assessment (EMA) during the 7 days the accelerometer is worn, via the MovisensXS app (Movisens, Karlsruhe, Germany). The measurement of momentary fatigue consists of three questions asking for feelings of tiredness, fitness and physical exhaustion and is based on the Short Fatigue Questionnaire.^43^ Momentary stress is also assessed with three questions, including two items from the PSS-10 questionnaire and one emotion item on nervous or stressed feelings. The questions were adapted for momentary use and previously used in EMA studies investigating momentary perceived stress.^44 45^ Prompts for momentary fatigue and momentary stress are sent six times per day, between 09:00 and 21:00 hours at random configuration, with at least 1 hour between prompts. The MovisensXS software is also used to prompt caffeine intake at 12:00 hours, 17:00 hours and 21:00 hours. Furthermore, a prompt is sent for alcohol use and timing of the main meal of the previous day using a start-of-day questionnaire. Furthermore, a sleep diary (Consensus Sleep Diary) is kept via the start-of-day questionnaire.^46^

Baseline characteristics regarding personal, disease-related and demographic measures are obtained from electronic health records and self-designed questionnaires. Attendance to the CR sessions and attendance to the intervention sessions are retrieved from electronic health records. A self-designed questionnaire is administered by a nurse via telephone to assess possible (serious) adverse events and other hospital visits.

Due to the nature of the study, blinding of participants and those giving the intervention is not possible. To minimise bias, objective measures of sleep and stress are also obtained using the GENEActiv accelerometer, as well as hair cortisol and cortisone.

### Sample size

The chosen sample size was based on expected differences in three somewhat related primary endpoints. We aim to detect an effect on at least one of these endpoints with a power of 80%. Therefore, for each comparison, a two-sided alpha of 0.017 was used.

Regarding sleep duration, the estimated mean±SD at 3-month measurement is 6.2±0.9 hours for the control group.^47^ We hypothesised that this value would be 10% higher for patients randomised to CR+RESST, equating to 6.8±0.9 hours at 3-month measurement. To detect this mean difference of 0.6 hours, 49 participants per group are needed. Regarding sleep quality, the expected mean PSQI score is 6.5±2.6 at 3-month follow-up for the control group, and 4.4±2.6 for the intervention group, based on research by Ghane *et al* and unpublished data from the OPTICARE-XL trial.^48^ To detect this mean difference in PSQI of 2.1 points between the control and intervention group, 68 participants are required. For the last primary outcome, perceived psychological stress, we expect a mean PSS-10 score of 15.4±8.2 is expected after CR alone, and a mean score of 11.1±8.2 for the intervention group.^19^ To detect this mean difference of 4.3 points, 156 participants are required. Thus, accounting for a minimum of 156 participants and a dropout of approximately 25%,^49 50^ we plan to include 200 participants in the study. This sample size will also allow us to take a number of covariates into account which may be associated with intervention effectiveness.

Drop-out and adherence will be continuously monitored throughout the study. If both remain lower than expected, participant inclusion may be stopped before reaching 200 participants to prevent unnecessary burdening.

### Statistical analysis

Descriptive statistics will be used to summarise the outcomes of the primary and secondary study parameters, as well as baseline characteristics (table 2).

Primary analyses will be conducted according to the intention-to-treat principle, but per-protocol analysis will be conducted to provide further insight into the effectiveness in adherent participants, as we aim to continue collecting data from non-adherent participants.

Linear mixed effect (LME) models will be used to study between-group differences in changes in primary and secondary endpoints between T0-T3m (primary analysis) and T0-T6m (secondary, explorative analysis). LME models can handle missing data and do not require data imputation.

To investigate whether parameters of diversity (age, sex and socioeconomic status) predict differences in changes in sleep and stress parameters between the intervention and control groups, an interaction term between randomly allocated treatment and the determinant of interest will be added to the LME model. If a significant interaction emerges, exploratory subgroup analyses may be performed.

A cost-effectiveness analysis will be performed using a healthcare and societal perspective according to the latest Dutch guidelines.^51^ A short-term time horizon of 6 months (total follow-up) will be analysed, as well as a lifetime time horizon, by using mathematical modelling for extrapolation beyond the follow-up period.^52^ Costs will include healthcare utilisation, intervention costs and productivity losses, while effects will be expressed in quality-adjusted life-years.

LMEs will also be used to examine the (bi)directional relationships between sleep, stress and lifestyle/health components. Lifestyle and health components include physical behaviour, diet intake and momentary fatigue. First, cross-sectional and longitudinal associations will be assessed using weekly averages of sleep/stress parameters and other lifestyle/health components. Additionally, day-to-day temporal relationships will be studied, while measurements on a certain moment may serve as a predictor for outcomes on a later moment. In these analyses, an outcome on a certain moment (eg, physical activity) may serve as the predictor for sleep/stress at a later moment during the day or on the following night/day, or conversely, sleep/stress at a certain moment may serve as the predictor for an outcome (eg, fatigue) during a later moment.

The statistical analyses will not be blinded due to limited study resources. However, the data analysis plan will be discussed with a biostatistician prior to conducting the analyses, and code will be checked by an independent researcher to prevent bias. Additionally, data and code will be made available in a data repository after study termination to promote transparency.

### Patient and public involvement

Patient representatives and CR professionals were involved in the initial design of the study (intervention). Additionally, a focus group interview with patients was held to evaluate the outcomes regarding completeness and relevance. The participants also provided input on the feasibility and burden of the measurements and participation in the study.

Patient meetings will be held yearly to provide input and to discuss results, where we will ensure a diverse sample of patients regarding age, sex, ethnicity, socioeconomic status and cardiac diagnosis. At the end of the study, approximately 10 interviews with patients and their relatives, and healthcare professionals will be conducted to collect information on experienced needs, barriers and enablers for further improvement and implementation of the intervention. These interviews may also guide interpretation of the differences between the intention-to-treat and per-protocol analyses, as barriers will be discussed. All interviews will be recorded, transcribed verbatim and analysed using the Consolidated Framework for Implementation Research.^53^

## Discussion

OPTICARE-RESST is a multicentre RCT aiming to include 200 CR participants experiencing poor sleep and/or psychological stress. To our knowledge, this is the first sizeable trial to evaluate a behavioural intervention simultaneously targeting sleep and stress in a cardiac population. This trial will provide insight into the (cost-)effectiveness of such a behavioural intervention on sleep and stress, the interrelatedness of sleep, stress and lifestyle factors such as physical behaviour, gather enablers and barriers for intervention implementation, and inform the optimisation of treatment for CR patients experiencing poor sleep and stress.

We expect that the newly developed RESST intervention will be effective in improving sleep and stress management as evidenced by improvements in self-reported and objective measures of sleep and stress in cardiac patients. In healthy adults, a meta-analysis demonstrated that similar interventions were effective in improving sleep quality and duration.^54^ Improvements were also seen in insomnia severity, sleep quality, fatigue, daytime sleepiness and QoL after receiving CBT-i or ACT(-i) in populations with comorbid conditions such as cardiac disease.^55^,^57^ The combined effectiveness of integrating CBT-i and ACT(-i) for improving insomnia symptoms, worry, anxiety and distress has been demonstrated in some small pilot studies in cancer- and CR patients,^58 59^ adding evidence to the suggestion that CBT and ACT may work in a complementary manner.^60^

Important strengths in the design are assessing sleep and stress both objectively and self-reported, which will provide insight into multiple facets of sleep and stress. Furthermore, the data collected with EMA gives insight into the daily lives of our participants and allows a more detailed perception of the day-to-day fluctuations that may occur, for example, in feelings of fatigue or stress, than questionnaires asking symptom recall over a previous period.^61^ By assessing in a momentary manner, recall bias is minimised. Combined with the simultaneously worn accelerometer, this allows for exploring the (bi)directional associations between eg, momentary experiences (eg, fatigue, stress or diet intake), and the 24 hours activity pattern of sleep and physical activity. As the data can be explored both cross-sectionally (averaged over a week) and momentarily across days (day-to-day), this will create more understanding of the interrelatedness of these factors. These insights, along with understanding how parameters such as age, sex and socioeconomic status affect intervention effectiveness, will help customise the intervention and promote equitable healthcare.

The study is limited by the relatively short follow-up time, which prevents evaluation of long-term effects on event recurrence or mortality. Furthermore, no blinding of participants or study personnel is possible due to the nature of the intervention. Lastly, there may be a bias in the objective measure for stress. As cortisol and cortisone are determined from the most proximal 1 cm of scalp hair, we very likely will obtain more samples from women than men, as a large number of the male CR participants might be (almost) bald.

During the project, end-users, such as patients, health practitioners and policy makers—including guideline developers and healthcare insurers—will be actively involved to develop a roadmap for further optimisation and implementation of the RESST intervention. Barriers and enablers, as well as participants’ perceptions and satisfaction, will be assessed. These barriers and enablers will inform the selection of implementation strategies, using the Expert Recommendation for Implementation Change taxonomy.^62^ This approach will maximise the real-world impact of the intervention and increase the likelihood of successful implementation if the intervention proves effective and cost-effective. If the intervention is not proven effective, the obtained insights will be essential to understand how the intervention can be improved to better meet patients’ needs.

In conclusion, we hypothesise that integrating a tailor-made behavioural intervention targeting sleep and stress into CR will significantly improve both outcomes. This is highly relevant given that over half of cardiac patients experience sleep and/or stress complaints, which negatively impact cardiovascular recovery and health, increase hospital readmissions and reduce QoL. If effective, this intervention has strong potential for implementation, offering a scalable solution to enhance patient recovery and long-term well-being.

## Ethics and dissemination

The study was designed according to the principles of the Declaration of Helsinki and the Medical Research Involving Human Subjects Act (WMO). The study was registered at ClinicalTrials.gov (NCT06505109) prior to trial initiation (10 July 2024; last update 16 June 2025). The study has been approved by the medical ethics committee of Erasmus MC (MEC-2024-0238). If protocol amendments are approved by the METC, all study personnel will be notified and retrained if necessary. At any point, the METC has the authority to (temporarily) halt participant inclusion. If harm arises to a participant due to the trial, the participant is able to use the collective trial insurance. Furthermore, participants can discontinue their participation at any given moment, without having to provide a reason.

An independent monitor will monitor all locations at least once to ensure protocol compliance and good clinical practice. To further ensure data validity, measures such as range checks have been put into place. All data is pseudonymised, with only the main researchers having access to the key. Data will be stored for 15 years after study completion, and biological samples will be destroyed immediately after analysis.

The findings will be disseminated through publications in peer-reviewed journals, presentations at academic conferences, and professional and patient publications. Pseudonymised data, a data dictionary and code will be made available to other researchers on request via a data repository.

## Supplementary material

10.1136/bmjopen-2026-117963 online supplemental file 1
